# Biodegradable polymer drug-eluting stents versus first-generation durable polymer drug-eluting stents: A systematic review and meta-analysis of 12 randomized controlled trials

**DOI:** 10.1097/MD.0000000000008878

**Published:** 2017-11-27

**Authors:** Pravesh Kumar Bundhun, Manish Pursun, Feng Huang

**Affiliations:** a Institute of Cardiovascular Diseases, the First Affiliated Hospital of Guangxi Medical University; b Guangxi Medical University; c Institute of Cardiovascular Diseases and Guangxi Key Laboratory Base of Precision Medicine in Cardio-cerebrovascular Diseases Control and Prevention, the First Affiliated Hospital of; Guangxi Medical University, Nanning, Guangxi, P. R. China.

**Keywords:** biodegradable polymer drug-eluting stents, durable polymer drug-eluting stents, long-term, major adverse cardiac events, randomized controlled trials, stent thrombosis

## Abstract

**Background:**

Even if drug-eluting stents (DES) showed beneficial effects in patients with coronary artery diseases (CADs), limitations have been observed with the first-generation durable polymer DES (DP-DES). Recently, biodegradable polymer DES (BP-DES) have been approved to be used as an alternative to DP-DES, with potential benefits. We aimed to systematically compare BP-DES with the first-generation DP-DES using a large number of randomized patients.

**Methods:**

Electronic databases were searched for randomized controlled trials (RCTs) comparing BP-DES with first-generation DP-DES. The main endpoints were the long-term (≥2 years) adverse clinical outcomes that were reported with these 2 types of DES. We calculated odds ratios (ORs) with 95% confidence intervals (CIs) and the analysis was carried out by RevMan 5.3 software.

**Results:**

Twelve trials with a total number of 13,480 patients (7730 and 5750 patients were treated by BP-DES and first-generation DP-DES, respectively) were included. During a long-term follow-up period of ≥2 years, mortality, myocardial infarction (MI), target lesion revascularization (TLR), and major adverse cardiac events (MACEs) were not significantly different between these 2 groups with OR: 0.84, 95% CI: 0.66–1.07; *P* = .16, *I*^2^ = 0%, OR: 1.01, 95% CI: 0.45–2.27; *P* = .98, *I*^2^ = 0%, OR: 0.91, 95% CI: 0.75–1.11; *P* = .37, *I*^2^ = 0% and OR: 0.86, 95% CI: 0.44–1.67; *P* = .65, *I*^2^ = 0%, respectively. Long-term total stent thrombosis (ST), definite ST, and probable ST were also not significantly different between BP-DES and the first-generation DP-DES with OR: 0.77, 95% CI: 0.50–1.18; *P* = .22, *I*^2^ = 0%, OR: 0.71, 95% CI: 0.43–1.18; *P* = .19, *I*^2^ = 0% and OR: 1.31, 95% CI: 0.56–3.08; *P* = .53, I^2^ = 6%, respectively.

**Conclusion:**

Long-term mortality, MI, TLR, MACEs, and ST were not significantly different between BP-DES and the first-generation DP-DES. However, the follow-up period was restricted to only 3 years in this analysis.

## 1 Introduction

Drug-eluting stents (DES) showed beneficial effects in patients with coronary artery diseases (CADs). However, limitations have been observed with the first-generation durable polymer DES (DP-DES), which are thick, and might be among the factors, which are associated with the initiation of vascular inflammatory reactions, therefore, contributing to the occurrence of late stent thrombosis (ST).

Recently, biodegradable polymer DES (BP-DES) have been approved to be used as an alternative to DP-DES, with potential benefits. BP-DES carry and control the drug, which is being released from DES during a specific period of time, and then erode and vanish from the vascular surface.

In other words, DES that are currently approved for use consist of a durable polymer (i.e., why they are called DP-DES), which is permanently attached to the stent even after the drug is eluted. Hence, the risk of ST becomes accountable when the polymer itself results in vascular inflammation or delay endothelialization and healing. However, in BP-DES, the polymer is removed and a bare metal like stent is left in order to reduce late ST.^[1]^

Insights from the 5 years follow-up of the randomized PAINT trial comparing very late outcomes of DES coated with biodegradable polymers releasing either paclitaxel or sirolimus showed that compared with bare metal stents (BMS), BP-DES were more effective in reducing major adverse cardiac events (MACEs) and reintervention without causing any increase in ST.^[2]^ In contrast, even if the first-generation DP-DES significantly decreased repeated revascularization when compared with BMS, they were associated with significantly higher incidence of very late ST.^[3]^

Nevertheless, BP-DES have seldom been compared with the first-generation DP-DES [sirolimus-eluting stents (SES) and paclitaxel-eluting stents (PES)] through meta-analyses. Hence, we aimed to systematically compare BP-DES with the first-generation DP-DES using a large number of randomized patients.

## 2 Methods

### 2.1 Data sources and search strategy

PubMed/Medline, EMBASE, and the Cochrane library were searched for randomized controlled trials (RCTs) comparing BP-DES with the first-generation DP-DES by typing the words or phrase “biodegradable polymer drug eluting stents and X” whereby “X” was replaced by either “sirolimus eluting stents or paclitaxel eluting stents.” Abbreviations such as “SES, PES and DES” were also used during the search process. To further enhance this search, the words “first generation DES” were also used. Reference lists of suitable articles were also searched for relevant trials. Only articles that were published in English were considered relevant during this search process.

### 2.2 Inclusion and exclusion criteria

Studies were included if

(1) They were published trials comparing BP-DES with the first-generation DP-DES (SES or PES);
(2) They reported adverse clinical outcomes as their endpoints.

Studies were excluded if

(1) They were non-RCTs (meta-analyses, observational studies, letter to editors);
(2) They did not report adverse clinical outcomes as their endpoints;
(3) They were duplicates;
(4) They were associated with the same trial.

### 2.3 Definitions, outcomes, and follow ups

The following adverse outcomes were analyzed:

(1) Mortality (all-cause mortality);
(2) Myocardial infarction (MI);
(3) Target vessel revascularization (TVR);
(4) Target lesion revascularization (TLR);
(5) MACEs;
(6) ST including total ST, definite ST, and probable ST. Total ST consisted of any type of ST, whereas definite and probable ST were defined according to the Academic Research Consortium (ARC).^[4]^

The main focus of this analysis was on the outcomes that were reported during a longer follow-up period of ≥2 years. However, outcomes were also analyzed during a mean follow-up period ranging from 8 months to 3 years and during a mid-term follow-up period of less than or equal to 1 year (≤1 year). Table 1^[5–16]^ summarized the outcomes that were reported among the trials with their corresponding follow-up periods and Table 2 summarized the types of participants and the antiplatelet regimens which were used.

**Table 1:**
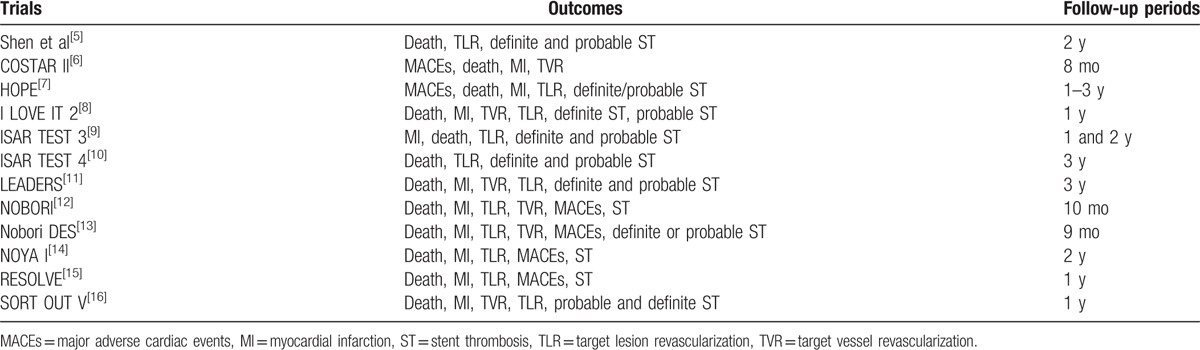
Reported outcomes and follow-up periods.

**Table 2:**
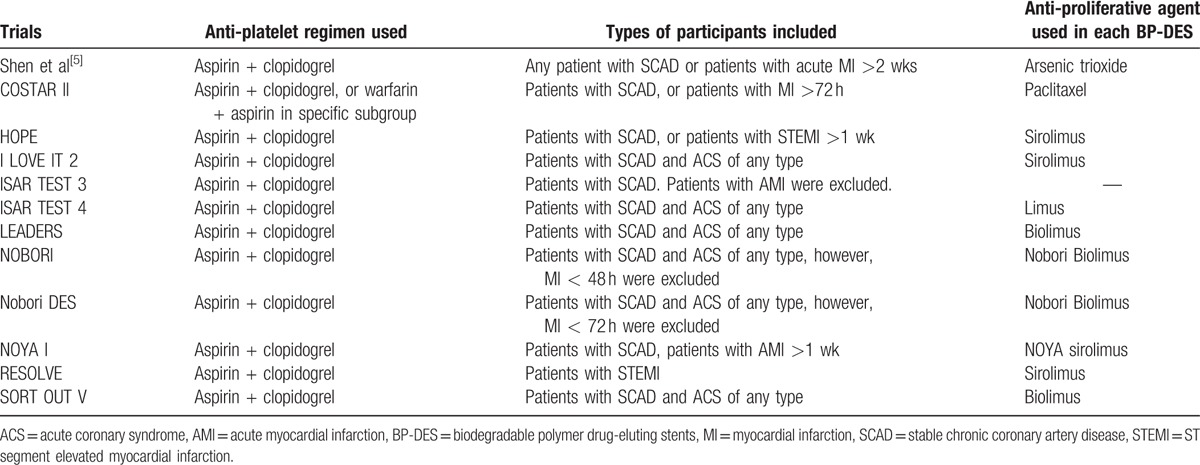
Types of patients included and the anti-platelet drugs that were used.

### 2.4 Data extraction and quality assessment

Two authors (PKB and MP) independently assessed the trials that were confirmed for this analysis. Information regarding the trial names, the year of publications, the total number of patients classified in the BP-DES, and first-generation DP-DES groups, respectively, relevant data associated with the baseline characteristics of the patients, the types of first generation DP-DES involved, the reported clinical outcomes with the corresponding number of events, and the follow-up periods were carefully extracted. Disagreements about including certain data were carefully discussed between these 2 authors. However, if they could not reach a consensus, disagreements were further resolved by the third author (FH). The bias risks were assessed using the recommendations approved by the Cochrane Collaborations^[17]^ whereby a grade ranging from A to E was allotted to the trials depending on the level of bias that was involved (Table 3).

**Table 3:**
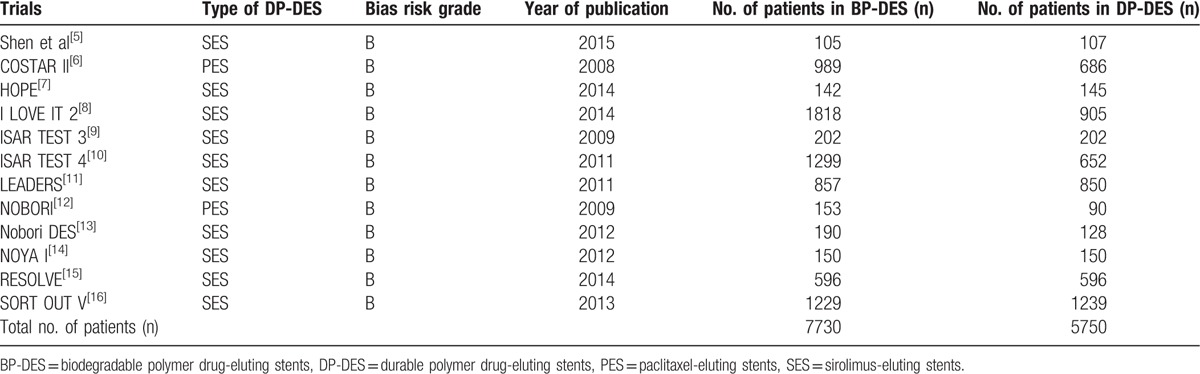
General features of the trials.

### 2.5 Methodological and statistical analysis

Recommendations of the PRISMA^[18]^ (Preferred Reporting Items for Systematic Reviews and Meta-Analyses) statement were followed. Heterogeneity across the trials was assessed using the Cochrane Q-statistic test whereby a *P* value of ≤.05 was considered statistically significant. Heterogeneity was also assessed by the *I*^2^-statistic test whereby a low percentage of *I*^2^ denoted a low heterogeneity while an increasing percentage denoted an increasing heterogeneity. If *I*^2^ was less than 50%, a fixed effects model was used. However, if *I*^2^ was more than 50%, a random effects model was used. We calculated odds ratios (OR) with 95% confidence intervals (CIs) and the pooled analyses were performed with RevMan 5.3 software.

Sensitivity analyses were carried out for the subgroups assessing the long-term follow up (≥2 years). The trials were excluded one by one and a new analysis was performed each time, to find out if there was any significant change in the results.

This current analysis did not involve a large number of trials; therefore, Egger or Begg tests were not considered necessary to assess publication bias. Instead, publication bias was visually estimated by assessing funnel plots that were obtained from the Revman software (normally for smaller volume of studies, funnel plots obtained from Revman are recommended to assess publication bias).

### 2.6 Ethical approval

Ethical approval was not necessary, as this was a systematic review and meta-analysis of randomized trials.

## 3 Results

### 3.1 Search outcomes

**Figure 1:**
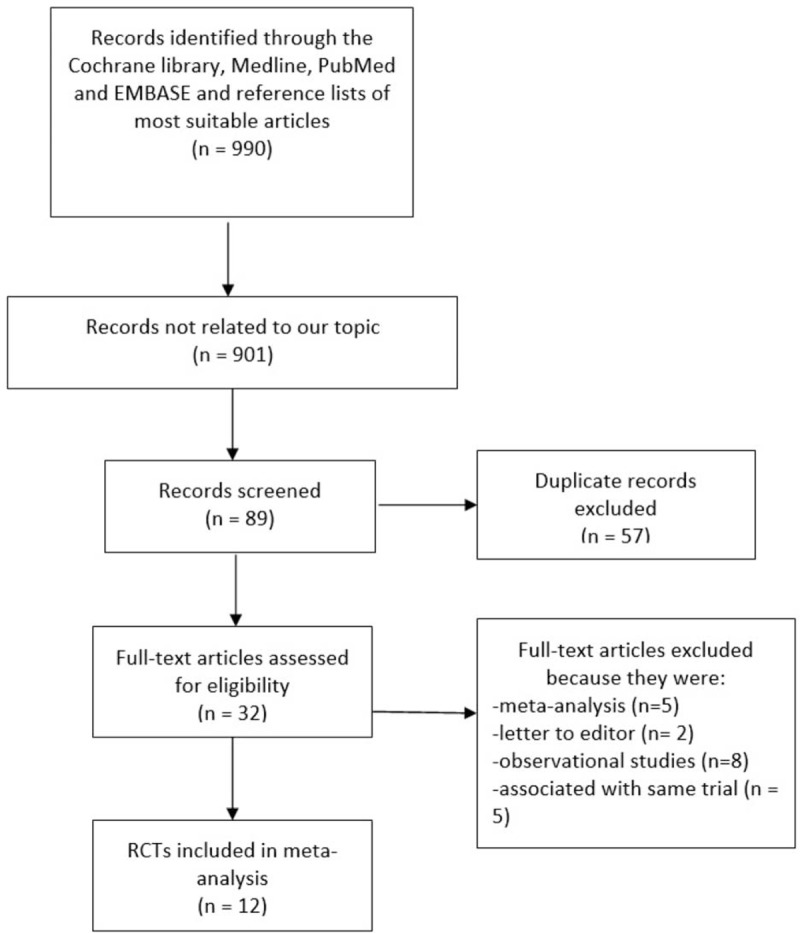
Flow diagram representing the study selection. Twelve trials that satisfied the inclusion and exclusion criteria of this study were finally included in this analysis.

Nine hundred sixty-five articles were obtained from electronic databases in addition to 25 more articles that were obtained from the reference lists of suitable studies. After a careful assessment of the titles and abstracts, 901 articles were eliminated, as they were not related to the topic of this research. A further 57 articles were eliminated, as they were duplicates. Thirty-two (32) full-text articles were assessed for eligibility. A further 20 articles were eliminated, as 8 articles were observational studies, 5 articles were meta-analyses, 2 articles were letter to editors, and 5 other articles involved the same trials. Finally, 12 trials^[5–16]^ were selected and included in this systematic review and meta-analysis as shown in Fig. 1.

### 3.2 General features of the trials

A total number of 13,480 patients (7730 patients who were treated with BP-DES and 5750 patients who were treated with the first-generation DP-DES) were included in this analysis. Further details about the total number of patients retrieved from each trial, the publication year, and the types of first-generation DP-DES (SES or PES) involved have been listed in Table 3.

### 3.3 Baseline characteristics of the patients

The baseline features of the patients have been summarized in Tables 4 and 5. The mean age of the patients who were treated by BP-DES ranged from 56.6 to 67.1 years, whereas the mean age of the patients who were treated by DP-DES ranged from 56.7 to 67.7 years. The number of male patients were above 66% in all the trials who were included. Trial HOPE had the least number of patients suffering from hypertension (54.9% vs 48.3% for BP-DES and DP-DES, respectively), RESOLVE trial had the least number of patients suffering from dyslipidemia (14.6% vs 12.6% for BP-DES and DP-DES, respectively), whereas the study by Shen et al^[7]^ had the highest number of patients suffering from dyslipidemia. Trial Nobori DES consisted of the largest number of patients with diabetes mellitus (38.7% vs 39.4% for BP-DES and DP-DES respectively). Most of the patients were being treated by elective PCI. According to these features, no significant differences were observed among patients who were implanted with BP-DES and the first-generation DP-DES.

**Table 4:**
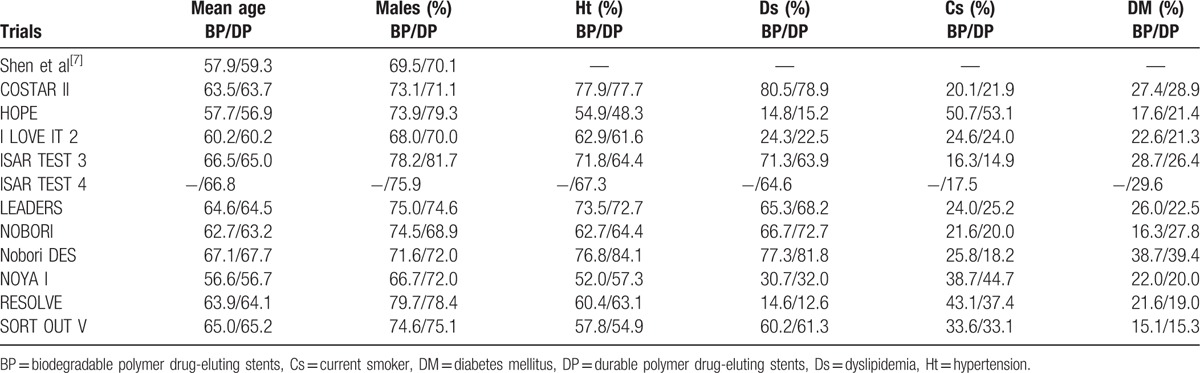
Baseline features of the patients (part A).

**Table 5:**
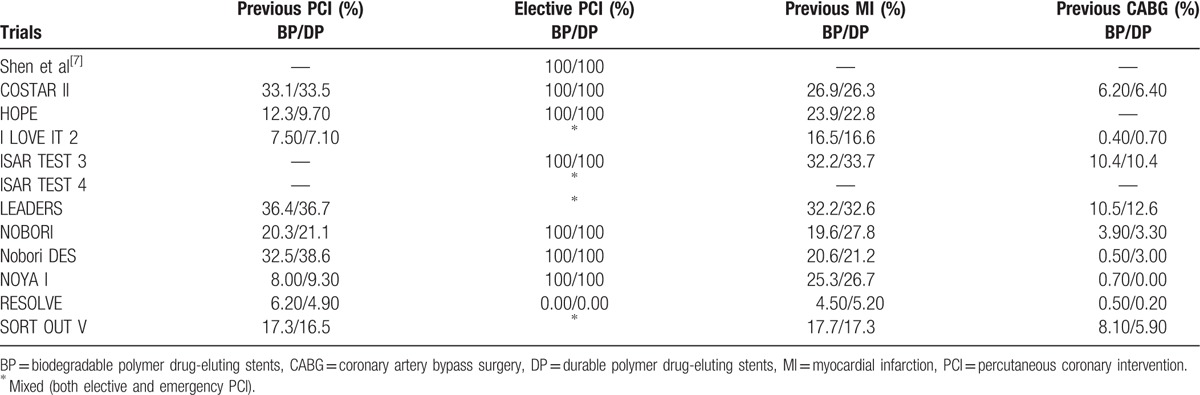
Baseline features of the patients (part B).

### 3.4 Adverse clinical outcomes that were reported during a follow-up period ranging from 8 months to 3 years

**Figure 2:**
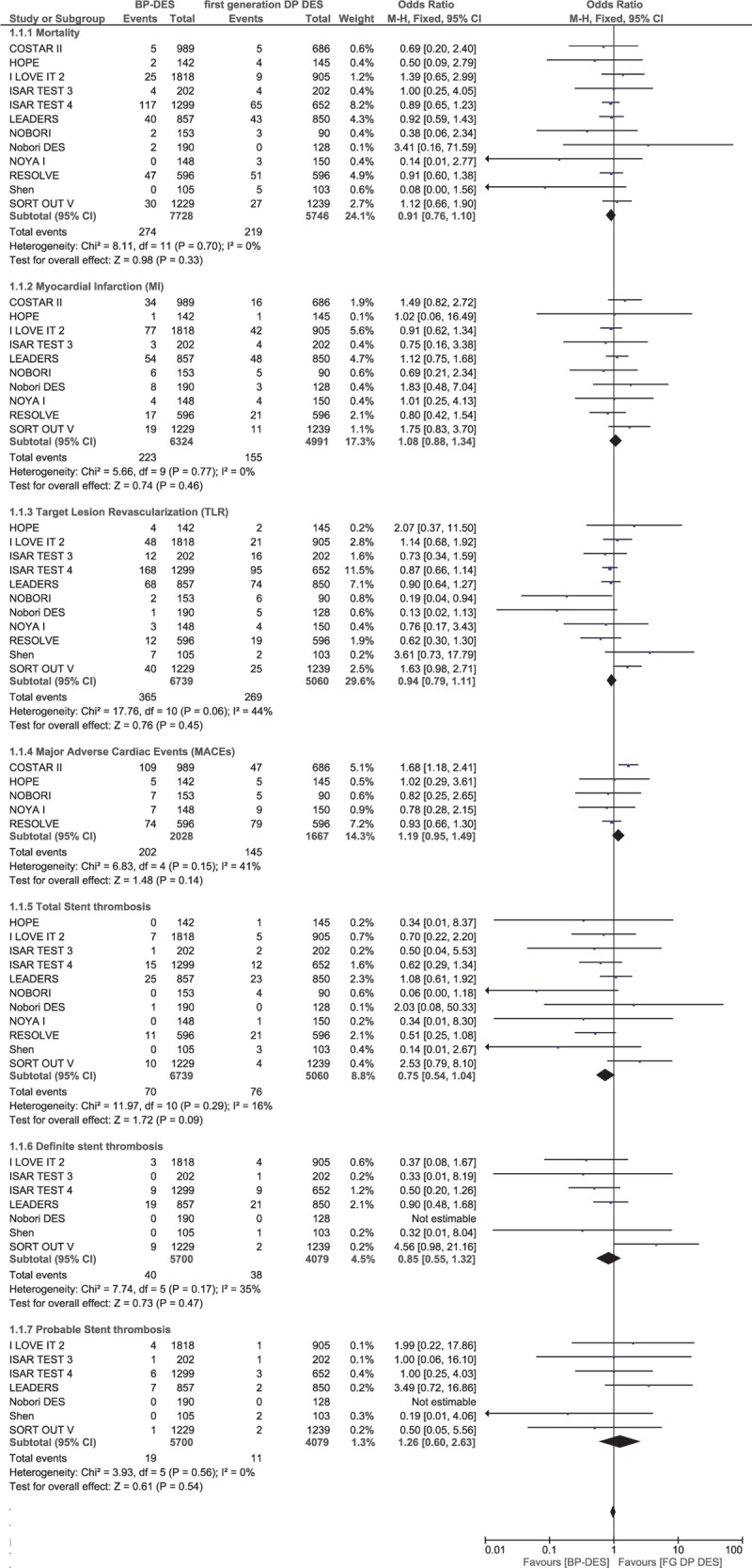
Comparing BP-DES with first-generation DP-DES during a follow-up period of 8 months to 3 years (part 1). No significant difference was observed between BP-DES and first-generation DP-DES as shown in the figure.

**Figure 3:**
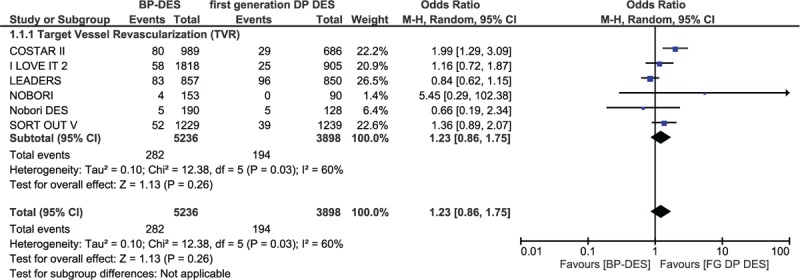
Comparing BP-DES with first-generation DP-DES during a follow-up period of 8 months to 3 years (part 2). No significant difference was observed between BP-DES and first-generation DP-DES as shown in the figure.

Results of this analysis showed that during a mean follow-up period ranging from 8 months to 3 years, mortality, MI, TLR, MACEs, and TVR were not significantly different between the BP-DES and first-generation DP-DES groups with OR: 0.91, 95% CI: 0.76–1.10; *P* = .33, *I*^2^ = 0%, OR: 1.08, 95% CI: 0.88–1.34; *P* = .46, *I*^2^ = 0%, OR: 0.94, 95% CI: 0.79–1.11; *P* = .45, *I*^2^ = 44%, OR: 1.19, 95% CI: 0.95–1.49; *P* = .14, *I*^2^ = 41% and OR: 1.23, 95% CI: 0.86–1.75; *P* = .26, *I*^2^ = 60%, respectively. Total ST, definite ST, and probable ST were also not significantly different between the BP-DES and first-generation DP-DES groups with OR: 0.75, 95% CI: 0.54–1.04; *P* = .09, *I*^2^ = 16%, OR: 0.85, 95% CI: 0.55–1.32; *P* = .47, *I*^2^ = 35% and OR: 1.26, 95% CI: 0.60–2.63; *P* = .54, *I*^2^ = 0%, respectively. These results have been illustrated in Figs. 2 and 3.

**Figure 4:**
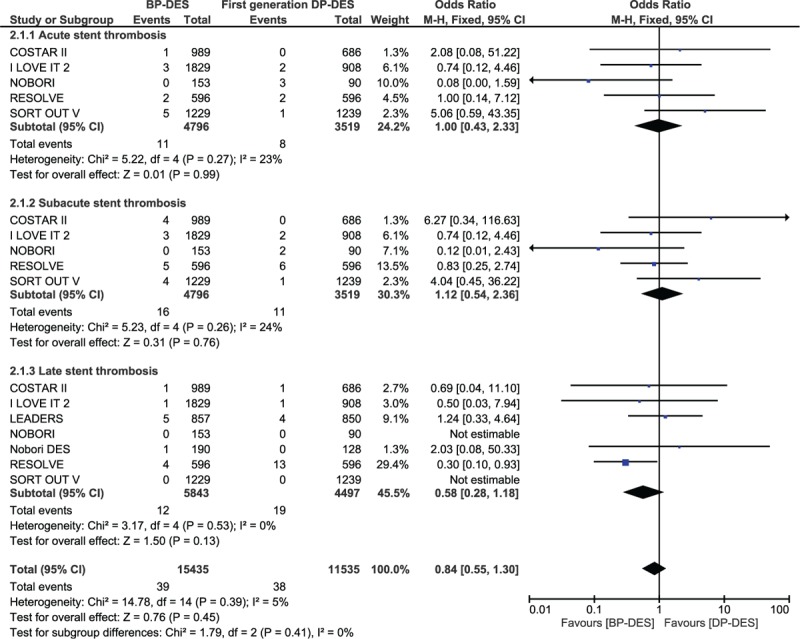
Comparing acute, subacute, and late stent thrombosis observed between BP-DES and DP-DES. No significant difference was observed between BP-DES and first-generation DP-DES, in terms of stent thrombosis as shown in the figure.

STs were further subdivided into acute ST, subacute ST, and late ST and then analyzed. Our results showed no significant difference observed with OR: 1.00, 95% CI: 0.43–2.33; *P* = .99, *I*^2^ = 23% for acute ST, OR: 1.12, 95% CI: 0.54–2.36; *P* = .76, *I*^2^ = 24% for subacute ST and OR: 0.58, 95% CI: 0.28–1.18; *P* = .13, *I*^2^ = 0% for late ST (Fig. 4).

**Figure 5:**
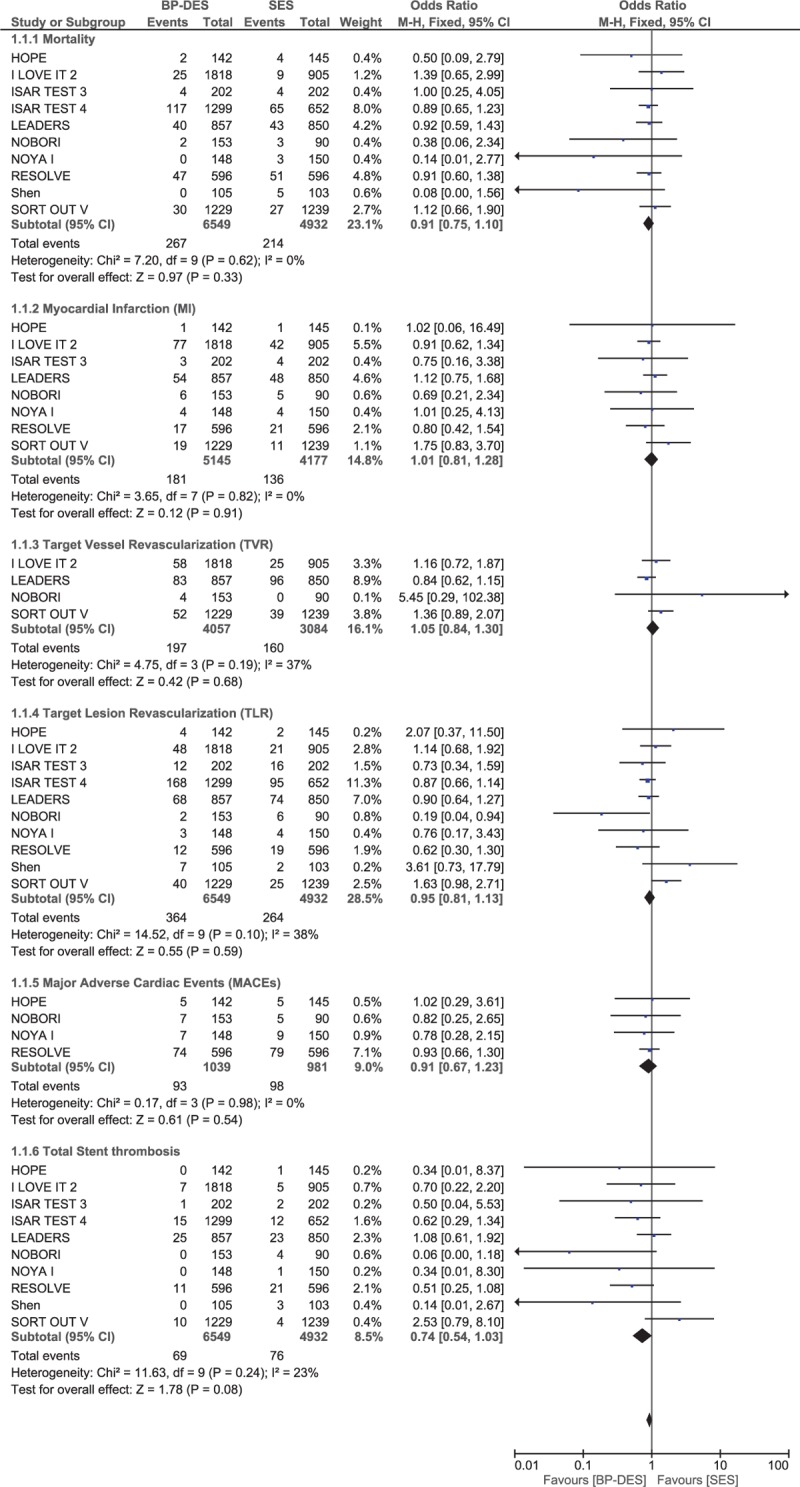
Comparing BP-DES with sirolimus-eluting stents (SES) during a follow-up period of 8 months to 3 years. Even if paclitaxel-eluting stents (PES) were excluded from the analysis, no significant difference was observed in the outcomes reported when comparing BP-DES with SES.

Another subgroup analysis was carried out, excluding patients with PES (COSTAR II and Nobori DES). When only patients implanted with SES were analyzed, there was still no significant change in the results. Mortality, MI, TVR, TLR, MACEs, and total ST were still not significantly different with BP-DES and SES with OR: 0.91, 95% CI: 0.75–1.10; *P* = .33, *I*^2^ = 0%, OR: 1.01, 95% CI: 0.81–1.28; *P* = .91, *I*^2^ = 0%, OR: 1.05, 95% CI: 0.84–1.30; *P* = .68, *I*^2^ = 37%, OR: 0.95, 95% CI: 0.81–1.13; *P* = .59, *I*^2^ = 38%, OR: 0.91, 95% CI: 0.67–1.23; *P* = .54, *I*^2^ = 0% and OR: 0.74, 95% CI: 0.54–1.03; *P* = .08, *I*^2^ = 23% respectively (Fig. 5).

### 3.5 Adverse clinical outcomes reported during the 1-year follow-up (mid-term)

**Figure 6:**
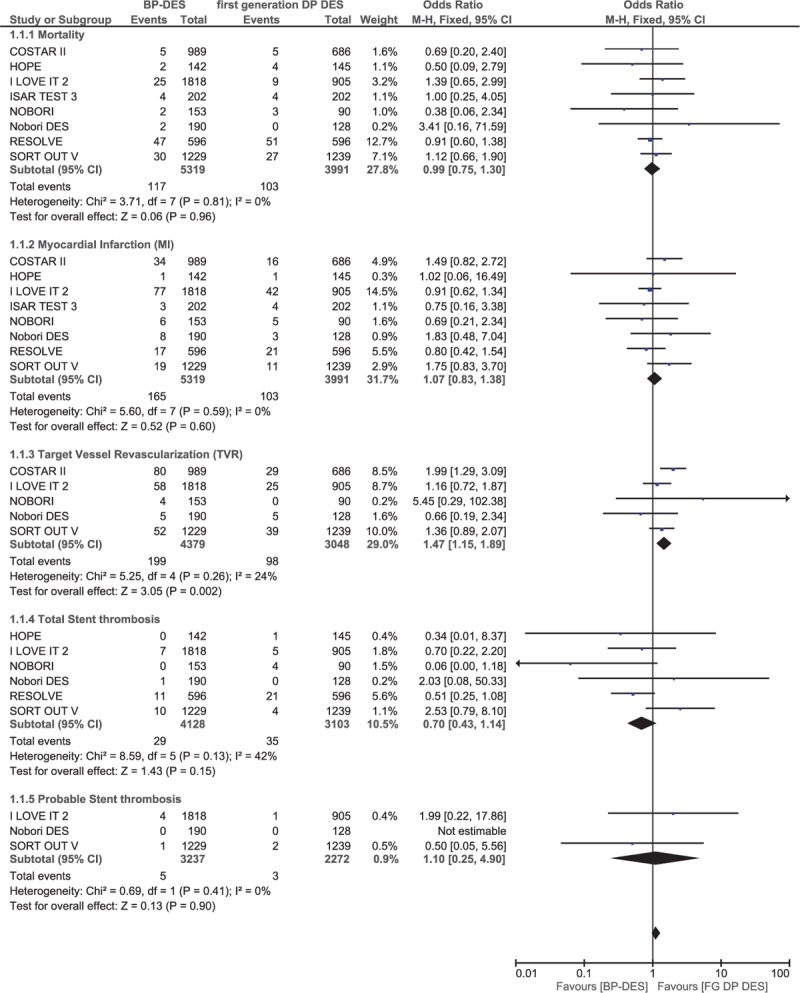
Comparing BP-DES with first-generation DP-DES at mid-term follow-up (part 1). No significant difference was observed between BP-DES and first-generation DP-DES as shown in the figure.

**Figure 7:**
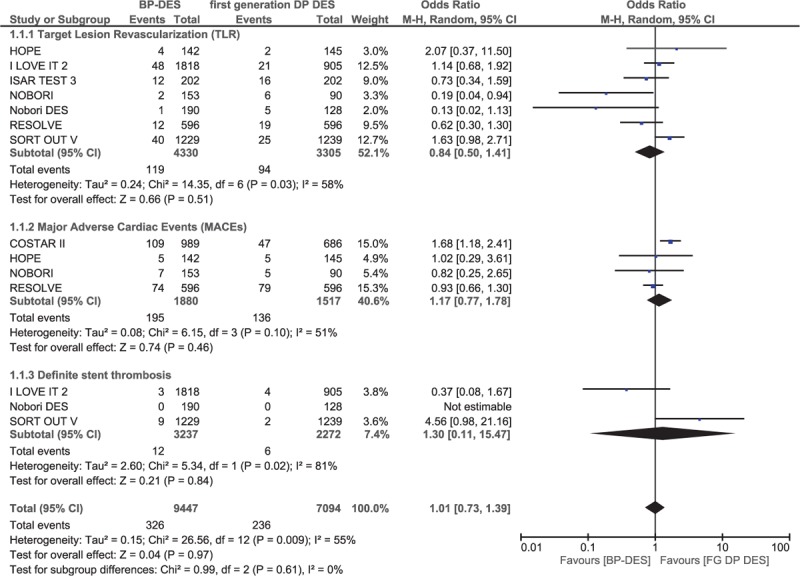
Comparing BP-DES with first-generation DP-DES at mid-term follow-up (part 2). No significant difference was observed between BP-DES and first-generation DP-DES as shown in the figure.

During a follow-up of 1 year or less, mortality and MI were not significantly different between the BP-DES and first-generation DP-DES with OR: 0.99, 95% CI: 0.75–1.30; *P* = .96, *I*^2^ = 0% and OR: 1.07, 95% CI: 0.83–1.38; *P* = .60, *I*^2^ = 60%, respectively. Total ST and probable ST were also not significantly different between these 2 groups with OR: 0.70, 95% CI: 0.43–1.14; *P* = .15, *I*^2^ = 0% and OR: 1.10, 95% CI: 0.25–4.90; *P* = .90, *I*^2^ = 0%, respectively. However, TVR significantly favored first-generation DP-DES with OR: 1.47, 95% CI: 1.15–1.87; *P* = .002, *I*^2^ = 24% (Fig. 6). Moreover, TLR, MACEs, and definite ST were also not significantly different with OR: 0.84, 95% CI: 0.50–1.41; *P* = .51, OR: 1.17, 95% CI: 0.77–1.78; *P* = .46, and OR: 1.30, 95% CI: 0.11–15.47; *P* = .84, respectively. These results have been summarized in Table 6 and illustrated in Fig. 7.

**Table 6:**
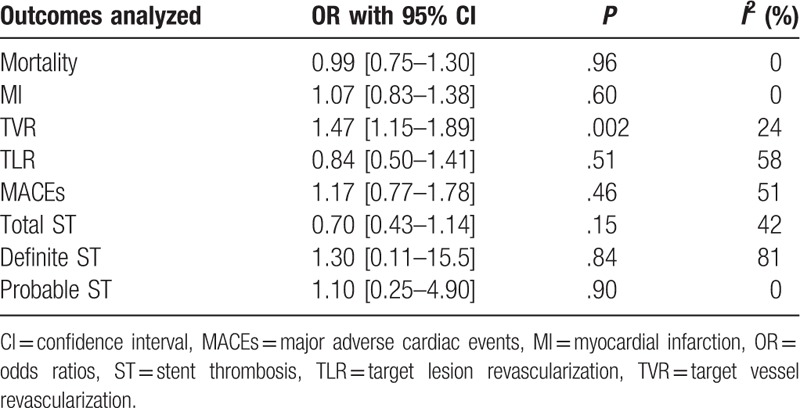
Results that were obtained during the 1-y follow-up (mid-term).

### 3.6 Adverse clinical outcomes reported at ≥2 years (long-term)

**Figure 8:**
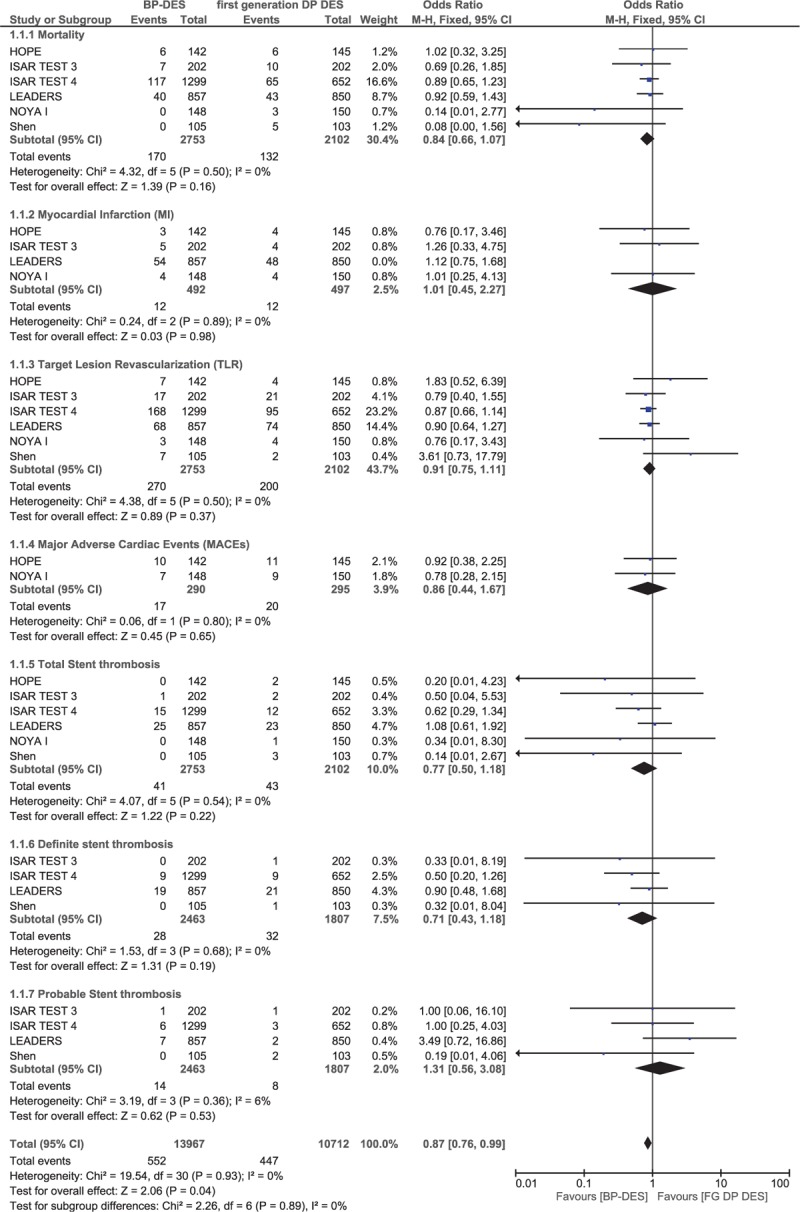
Comparing BP-DES with first-generation DP-DES during a long-term follow-up period (≥2 years). Even during a long-term follow-up period, no significant difference was observed between BP-DES and first-generation DP-DES as shown in the figure.

Outcomes were also analyzed during a long-term follow-up period of 2 or more years (involving 4855 patients). The results showed that mortality, MI, TLR, and MACEs were still not significantly different between these 2 groups with OR: 0.84, 95% CI: 0.66–1.07; *P* = .16, *I*^2^ = 0%, OR: 1.01, 95% CI: 0.45–2.27; *P* = .98, *I*^2^ = 0%, OR: 0.91, 95% CI: 0.75–1.11; *P* = .37, *I*^2^ = 0%, and OR: 0.86, 95% CI: 0.44–1.67; *P* = .65, *I*^2^ = 0%, respectively. Long-term total ST, definite ST, and probable ST were also not significantly different between BP-DES and first-generation DP-DES with OR: 0.77, 95% CI: 0.50–1.18; *P* = .22, *I*^2^ = 0%, OR: 0.71, 95% CI: 0.43–1.18; *P* = .19, *I*^2^ = 0%, and OR: 1.31, 95% CI: 0.56–3.08; *P* = .53, *I*^2^ = 6% respectively. These results have been summarized in Table 7 and represented in Fig. 8.

**Table 7:**
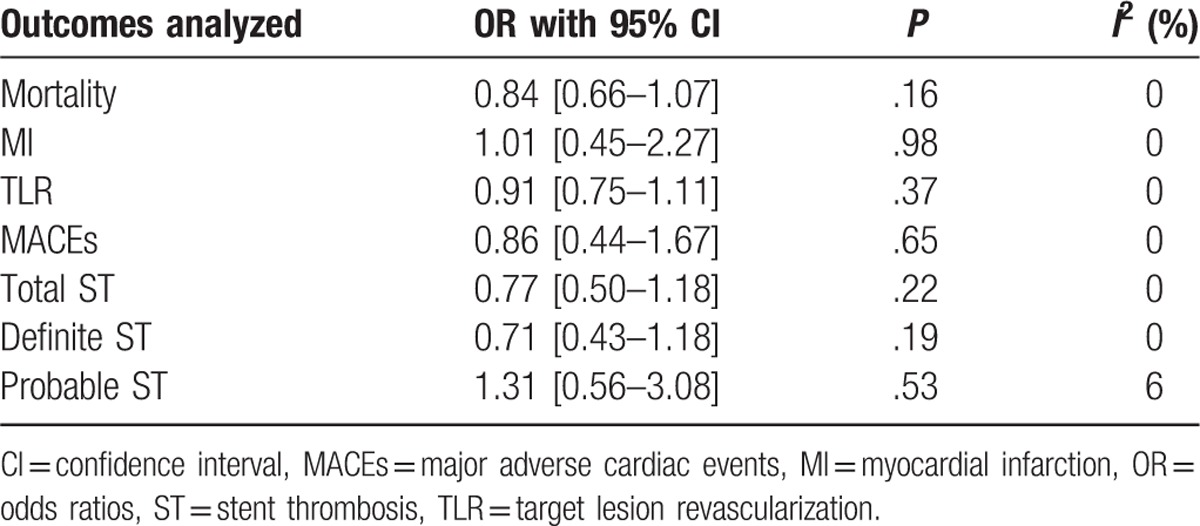
Results that were obtained at ≥2 y follow-up (long-term).

### 3.7 Sensitivity analysis

For all of the above analyses, sensitivity analyses were carried out and yielded consistent results. For the long-term follow-up (≥2 years), all the trials were excluded one by one and a new analysis was carried out each time, to find out whether there was any significant change in the results. However, no significant difference was observed and consistent results were obtained.

When trial HOPE was excluded during the long-term (≥2 years) follow-up, mortality, MI, TLR, and total ST were still not significantly different with OR: 0.88, 95% CI: 0.69–1.14; *P* = .34, OR: 1.09, 95% CI: 0.75–1.58; *P* = .66, OR: 0.87, 95% CI: 0.71–1.07; *P* = .18, and OR: 0.85, 95% CI: 0.55–1.33; *P* = .48, respectively. When trial ISAR TEST 3 was excluded, no significant difference was observed. The same thing was observed when trial ISAR TEST 4 was excluded. Mortality, TLR, and total ST were not significantly different with OR: 0.84, 95% CI: 0.56–1.26; *P* = .40, OR: 0.89, 95% CI: 0.66–1.21; *P* = .47, and OR: 0.96, 95% CI: 0.56–1.64; *P* = .87, respectively. Even when trial LEADERS was excluded, similar results were obtained.

**Figure 9:**
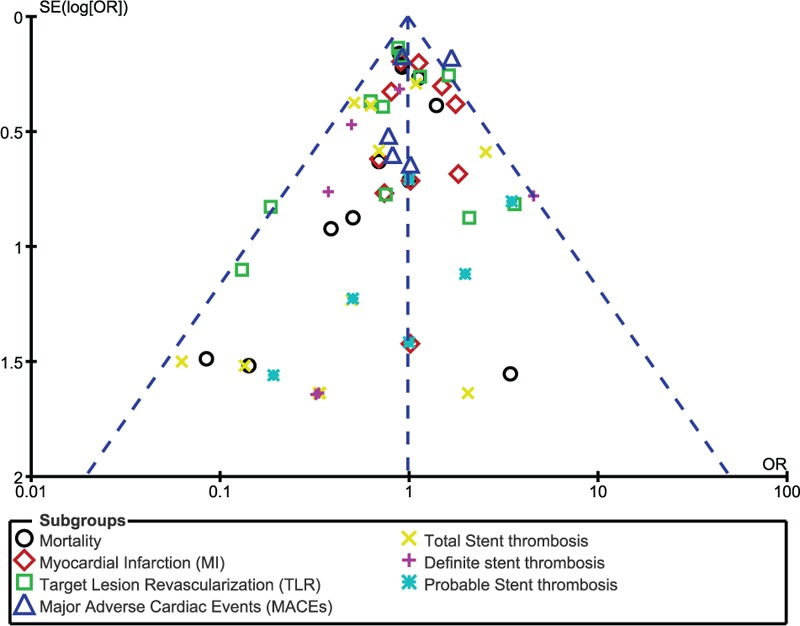
Funnel plot representing publication bias. Publication bias was visually assessed using funnel plots obtained from RevMan. A very low evidence of publication bias was observed among all the trials included in this analysis. Symmetrical funnel plots with a clearly defined center showed evidence of low bias.

**Figure 10:**
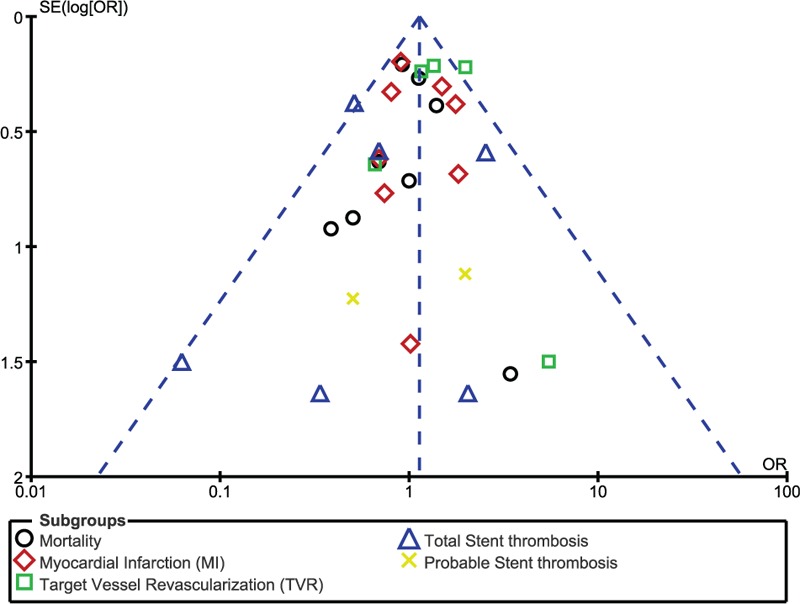
Funnel plot representing publication bias. Publication bias was visually assessed using funnel plots obtained from RevMan. A very low evidence of publication bias was observed among all the trials included in this analysis. Symmetrical funnel plots with a clearly defined center showed evidence of low bias.

**Figure 11:**
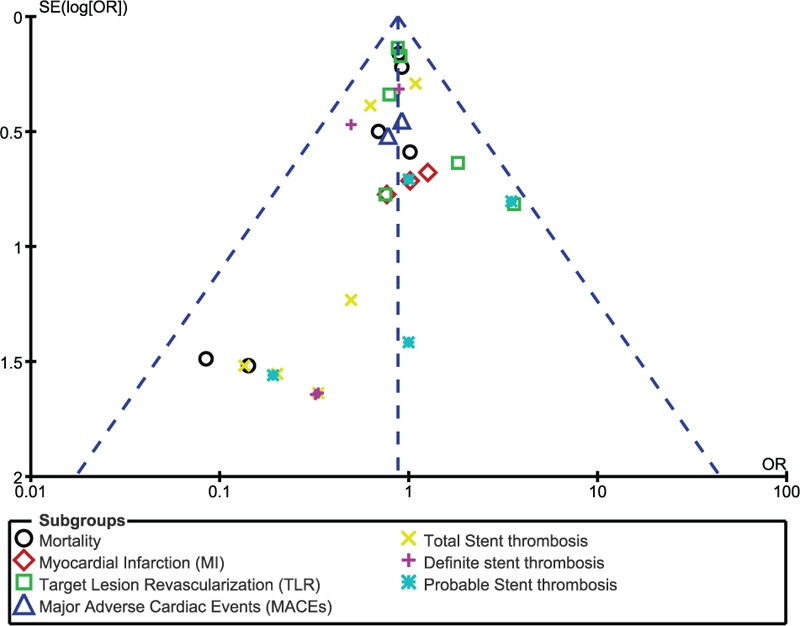
Funnel plot representing publication bias. Publication bias was visually assessed using funnel plots obtained from RevMan. A very low evidence of publication bias was observed among all the trials included in this analysis. Symmetrical funnel plots with a clearly defined center showed evidence of low bias.

**Figure 12:**
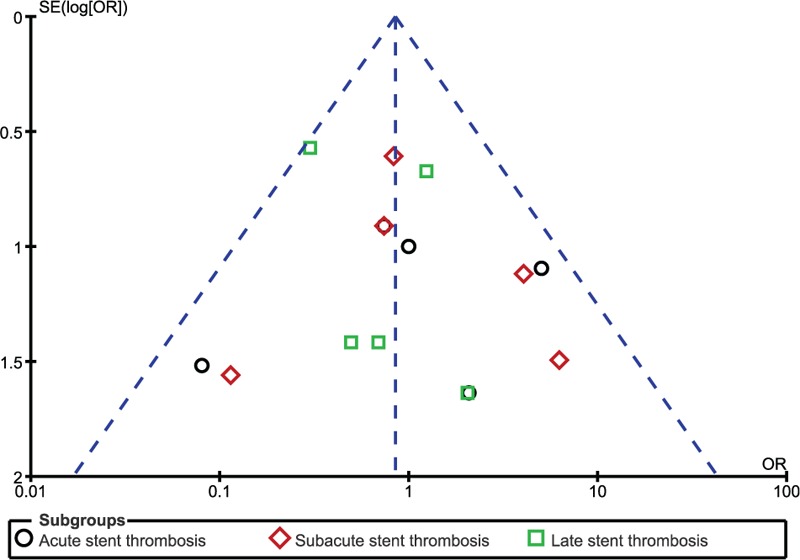
Funnel plot representing publication bias. Publication bias was visually assessed using funnel plots obtained from RevMan. A very low evidence of publication bias was observed among all the trials included in this analysis. Symmetrical funnel plots with a clearly defined center showed evidence of low bias.

In addition, on the baiss of a visual inspection of the funnel plots obtained, there has been very little evidence of publication bias for the included studies that assessed all clinical endpoints (mortality, MI, TVR, TLR, MACEs, and ST) (Figs. 9–12).

## 4 Discussion

In this analysis, we aimed to show whether the long-term adverse outcomes, which were associated with BP-DES, were significantly different when compared with those associated with first-generation DP-DES.

Current results showed that during a longer follow-up period, mortality, MI, MACEs, and TLR were not significantly different between BP-DES and the first-generation DES. Total ST, definite ST, and probable ST were also not significantly different between BP-DES and durable polymer SES or PES.

A recent meta-analysis involving 3 randomized trials with SES and everolimus-eluting stents (EES), respectively, and 1 trial with PES, comparing BP-DES with DP-DES, also showed that MACEs were not significantly different between these 2 groups, but however, BP-DES were associated with a significantly lower rate of very late ST than DP-DES.^[19]^ Another meta-analysis involving 15 randomized trials comparing BP-DES with DP-DES during a mean follow-up period of 20.6 months showed that both types of stents were equally effective and safe to use.^[20]^ However, in exception to the inclusion of SES and PES, it also involved 4 trials with EES. In addition, the authors suggested further long-term studies to confirm their results.

Also, the updated meta-analysis by Wang et al^[21]^ also showed similar clinical benefits between BP-DES and first-generation DP-DES. However, as only 5 randomized trials were included, the authors concluded that the incidence of very late ST should be confirmed in other future studies. Even though a total number of 12 randomized trials were included in this current analysis, our results were similar with that of the study published by Wang et al.^[21]^ Excluding data that were obtained from randomized trials, even an observational study comparing BP-DES with DP-PES showed comparable adverse outcomes (TVR, MACEs, ST) between these 2 types of stents during a 1-year follow-up period.^[22]^ The LEADERS trial involving 1707 patients from 10 centers showed BP-DES to be noninferior to SES in terms of the primary endpoints at 5 years; however, BP-DES were associated with a significantly lower rate of very late (from 1 year to 5 years) ST than DP-SES.^[23]^

Nevertheless, other studies showed results that deviated partly or completely from this current analysis. For example, the meta-analysis published by Lv et al showed BP-DES to be safe, efficient, and exhibiting superior performance compared with DP-DES in terms of very late ST.^[24]^ However, their study involved all types of DP-DES, whereas this current analysis only involved first-generation DP-DES. Another study showed BP-DES to be more effective in reducing MACEs and ST than DP-DES during the long term.^[25]^ Even the study published by Zhu et al^[26]^ showed BP-DES to be associated with a lower rate of very late ST. However, the authors suggested further studies to confirm their findings. Nevertheless, even if BP-DES showed to be more effective than DP-DES, this efficacy was more visible only with SES.^[27]^ But in this current study, even when SES were separately compared with BP-DES, no significant difference was observed.

However, these adverse outcomes might not always be dependent on the types of stents that were implanted. Several other studies have shown that the types of patients who were involved,^[28,29]^ age of the patients, the comorbidities, and complications which were present before or following PCI,^[30–32]^ the types of anti-platelets that were used and the duration period of DAPT,^[33,34]^ the dosage of aspirin that was used,^[35]^ could all contribute to and have a great impact on the adverse clinical outcomes following PCI.

Several studies have also shown bleeding risk to be affected by the duration of DAPT use. A decrease in major bleeding, without any increase in mortality or ST, has systematically been demonstrated with a shorter duration of DAPT (≤6 months).^[33]^ In patients who were implanted with second-generation DES, abbreviated DAPT duration (≤6 months) was considered adequately protective with lower bleeding events.^[36]^ One of the possible advantages of BP-DES is the decreased risk of late ST, hence, requiring a shorter duration of DAPT use, which is associated with less bleeding. In the RESOLVE trial, only 7 out of the 596 patients who were implanted with BP-DES reported major bleeding compared with 9 out of 596 patients who were implanted with DP-DES.^[15]^

This current analysis involved only BP-DES and the first-generation DP-DES. A large number of randomized patients were included and this study also satisfied all the criteria suggested for a well-conducted meta-analysis in terms of robust data, low heterogeneity among the subgroups analyzing the long-term outcomes, low risk of bias, and highly conducted statistical analyses, and hence could be used in clinical medicine to predict prognosis in patients who were implanted with either BP-DES or first-generation DP-DES.

### 4.1 Limitations

This analysis also has limitations. First of all, due to the limited number of patients, the results of this analysis might be restricted in certain ways. Moreover, the long-term follow-up period was restricted to only 3 years. Further studies with longer follow-up periods would have been more interesting. Unfortunately, data with even longer follow-up periods were not available. In addition, MACEs were reported in only a few trials. Therefore, only a few trials were included in the subgroup analysis of long-term MACEs. This could also represent another limitation of this analysis. Also, the subgroup analyzing total ST included a combination of different types of ST with different definitions. However, heterogeneity was not observed, as most STs, which were reported, were definite and probable ST as defined by the ARC classification. In addition, the inclusion of a variety of patients with stable chronic CAD, unstable CAD (ST segment elevation myocardial infarction and non-ST segment elevated myocardial infarction) could also represent a limitation of this study. In addition, the duration of dual anti-platelet agents might also have had an effect on the results that were obtained.

## 5 Conclusion

Long-term mortality, MI, TLR, MACEs, and ST were not significantly different between BP-DES and the first-generation DP-DES. However, the follow-up period was restricted to only 3 years in this analysis.
